# Biosacetalin (1,1-Diethoxyethane) Prolongs Survival and Alleviates Cachexia in the NSG Mice Bearing Neuroblastoma SH-SY5Y Cells

**DOI:** 10.3390/antiox15040521

**Published:** 2026-04-21

**Authors:** Dhiraj Kumar Sah, Thang Nguyen Huu, Jin Myung Choi, Vu Hoang Trinh, Hyun Joong Yoon, Seung-Rock Lee

**Affiliations:** 1 Department of Biochemistry, Research Center for Aging and Geriatrics, Research Institute of Medical Sciences, Chonnam National University Medical School, Gwangju 61469, Republic of Korea; 206847@jnu.ac.kr (T.N.H.); trinhoangvu@jnu.ac.kr (V.H.T.); hjms0320@hanmail.net (H.J.Y.); 2 Department of Biomedical Sciences, Research Center for Aging and Geriatrics, Research Institute of Medical Sciences, Chonnam National University Medical School, Gwangju 61469, Republic of Korea; 3 Luxanima Inc., Room 102, 12-55, Sandan-gil, Hwasun-eup, Hwasun-gun 58128, Republic of Korea; choijm1@naver.com; 4 Department of Oncology, Pham Ngoc Thach University of Medicine, Ho Chi Minh City 700000, Vietnam; 5 Department of Medical Sciences, Pham Ngoc Thach University of Medicine, Ho Chi Minh City 700000, Vietnam

**Keywords:** 1,1-diethoxyethane (biosacetalin), AMPK, apoptosis, anti-Warburg effect, survival, cancer cachexia, neuroblastoma, SH-SY5Y, NSG mice

## Abstract

Neuroblastoma remains a formidable pediatric malignancy characterized by profound metabolic plasticity and limited therapeutic responsiveness in high-risk disease. Emerging evidence positions the interplay between Reactive Oxygen Species (ROS) and the metabolic sentinel AMP-activated protein kinase (AMPK) as a critical regulator of tumor metabolic stress and apoptotic susceptibility, with additional implications in the systemic pathology of Cancer Cachexia. Building on our previous work demonstrating that 1,1-Diethoxyethane (1,1-DEE; Biosacetalin), a volatile aroma compound inhibits mitochondrial complex I, induces ROS production, and activates AMPK-PGC1α-mediated mitochondrial biogenesis accompanying enhancement of aerobic respiration, leading to anti-Warburg effect. We identify 1,1-DEE as a previously unrecognized metabolic modulator with potent antitumor activity. 1,1-DEE triggers ROS-induced AMPK activation, leading to apoptotic elimination of neuroblastoma cells (SH-SY5Y), robust suppression of tumor growth, and significant prolongation of survival (median survival 77 days) in tumor-bearing NSG mice. Strikingly, 1,1-DEE simultaneously alleviates cancer-associated cachexia by preserving body weight. Mechanistically, our findings reveal a ROS–AMPK–centered signaling axis through which 1,1-DEE integrates tumor-selective cytotoxicity with systemic metabolic protection, highlighting a unified therapeutic strategy for targeting both tumor progression and cachexia in neuroblastoma.

## 1. Introduction

Neuroblastoma is among the most aggressive pediatric malignancies, arising from aberrant differentiation of neural crest–derived progenitor cells within the developing sympathetic nervous system and accounting for a substantial fraction of childhood cancer mortality [1,2,3]. Notwithstanding substantial progress in contemporary multimodal therapeutic strategies—including dose-intensified chemotherapy, immunotherapeutic regimens, and the integration of emerging molecularly targeted agents within clinical trials—the prognosis for children afflicted with high-risk neuroblastoma remains discouragingly limited, with long-term survival rates generally confined to approximately 50–70% [4,5]. The persistently dismal clinical outcomes associated with this disease underscore a critical and unmet need to delineate the molecular determinants that orchestrate tumor initiation, progression, and therapeutic refractoriness.

Accumulating evidence identifies Reactive Oxygen Species (ROS) as critical determinants of tumor cell fate. While moderate ROS levels can sustain oncogenic signaling and metabolic adaptation, excessive oxidative stress perturbs redox homeostasis, induces mitochondrial dysfunction, and ultimately triggers Apoptosis [6,7,8]. Central to this stress-responsive network is AMP-activated protein kinase (AMPK), a master metabolic sensor that integrates energetic and oxidative cues to reprogram cellular metabolism and regulate survival–death decisions in a context-dependent manner. Emerging evidence further implicates dysregulation of the ROS–AMPK axis in the pathogenesis of Cancer Cachexia, a multifactorial wasting syndrome characterized by progressive loss of skeletal muscle and adipose tissue, systemic inflammation, and profound metabolic imbalance [9,10,11]. Collectively, these findings position ROS–AMPK signaling as a critical nexus linking tumor metabolic vulnerability, apoptotic signaling, and cachexia-associated systemic dysfunction. Elucidation of this molecular circuitry may therefore provide a strategic framework for the development of mechanism-driven therapeutic interventions capable of simultaneously inducing tumor-selective apoptosis and alleviating cachexia-related metabolic deterioration in Neuroblastoma.

Building upon this conceptual framework, we investigated 1,1-Diethoxyethane (1,1-DEE), a volatile aroma compound present in Sherry wine and distinguished by its characteristic fruity profile. Our previous studies revealed that 1,1-DEE exerts profound metabolic regulatory effects, including transient inhibition of mitochondrial respiration and glycolysis, increase of ROS production, robust activation of AMPK and induction of PGC-1α-TFAM mediated mitochondrial biogenesis. In addition 1,1-DEE was also shown to enhance insulin sensitivity and ameliorate obesity and dyslipidemia in the mice fed a high-fat diet [12,13,14,15]. Notably, these metabolic adaptations were accompanied by enhanced survival and physiological resilience in *Caenorhabditis elegans* and *Sprague–Dawley rat*, highlighting the compound’s systemic bioactivity and translational promise [16,17]. In the present study, we explored the therapeutic relevance of 1,1-DEE in Neuroblastoma and identified a previously unrecognized mechanism whereby ROS-driven AMPK activation promotes tumor-selective apoptosis while concurrently mitigating metabolic disturbances associated with Cancer Cachexia.

## 2. Materials and Methods

### 2.1. Cell Viability Assay

SH-SY5Y cells were plated in 96-well plates at a density of 2 × 10^4^ cells per well (100 µL) in RPMI medium supplemented with 10% fetal bovine serum (FBS) and 1% penicillin-streptomycin. On the following day, various concentrations of 1,1-DEE (Sigma-Aldrich, St. Louis, MO, USA) and its 1,2-DEE (TCI; Tokyo Chemical Industry Co., Ltd., Tokyo, Japan) were added to each well in serial dilutions (total volume of 120 µL). The cells were incubated for 24 h, after which 10 µL of MTT (DoGenBio Co., Ltd., Seoul, Republic of Korea) was added to each well and incubated at 37 °C for 2 h. Cell viability was evaluated by measuring the absorbance at 450 nm using a microplate spectrophotometer (Epoch; BioTek, Winooski, VT, USA).

### 2.2. Cell Culture

SHSY-5Y (human neuroblastoma, KCLB NO:22266) cells were purchased from the Korean Cell Line Bank (Korean Cell Line Bank, Korean Cell Line Research Foundation, Seoul National University Samsung Cancer Research Building (Yeongeon-dong), 101, Daehak-ro, Jongno-gu, Seoul, 03080, Republic of Korea) and cultured in growth medium containing RPMI 1640 supplemented with 10% FBS and 1% penicillin-streptomycin, at 37 °C in a humidified incubator with 5% CO_2_.

### 2.3. Western Blotting

SH-SY5Y cells (5 × 10^5^ cells/mL) were cultured in 60 × 15 mm culture dishes (SPL Life Sciences, Pocheon, Republic of Korea). Protein extraction was performed using Pro-PREPTM protein extraction solution (iNtRON Biotechnology, Seongnam, Republic of Korea), adding 150 µL per plate. Polyvinylidene fluoride (PVDF) membranes and Western Chemiluminescent HRP Substrate were purchased from Millipore Corporation (Billerica, MA, USA). Total protein (30 μg) was separated by 10% sodium dodecyl sulfate-polyacrylamide gel electrophoresis (SDS-PAGE) and transferred to PVDF membranes. The membranes were blocked for 1–2 h with 0.1% Tween-20 in TBST containing 5% skim milk, followed by overnight incubation with primary antibodies (1:1000) in TBST at 4 °C. After three washes with TBST (10 min each), the membranes were incubated with horseradish peroxidase-conjugated secondary antibody (1:2000) for the detection of immunoreactive proteins via chemiluminescence. Antibodies used included anti-phospho-AMPK (#2535S), anti-AMPK (#2532S), and anti-β-actin (#5125S) from Cell Signaling Technology (Danvers, MA, USA). Total protein levels were assessed by stripping the blotted membranes using Restore^TM^ Western Blot Stripping Buffer (Thermo Fisher Scientific, Rockford, IL, USA) at 56 °C for 30 min.

### 2.4. Annexin V-FITC Analysis of Cell Apoptosis

Apoptosis in SH-SY5Y cells was assessed using an Annexin V-FITC apoptosis detection kit (BD Pharmingen, San Diego, CA, USA) following the manufacturer’s protocol. Cells were seeded in 6-well plates at a density of 2.0 × 10^5^ cells per well in RPMI medium with 10% FBS and incubated for 24 h. Following treatment with various concentrations of 1,1-DEE for 24 h at 37 °C, the cells were harvested, pelleted, and resuspended in 400 µL of binding buffer. The cells were stained with 5 µL of Annexin V-FITC and 10 µL of PI for 15 min in the dark. Apoptotic cells were analyzed by flow cytometry using BD FACSCalibur (BD Biosciences, San Jose, CA, USA). Data were analyzed with FlowJo™ software (BD Biosciences, San Jose, CA, USA).

### 2.5. Measurement of ROS Production

The level of intracellular H_2_O_2_ was measured using 2′,7′-dichlorodihydrofluorescein diacetate (DCFDA; Molecular Probes, Eugene, OR, USA). In brief, SH-SY5Y cells were cultured to 80% confluency in RPMI medium supplemented with 10% FBS. To assess the effects of 1,1-DEE on ROS production, the cells were pretreated with 2 mM NAC 1 h before exposure to 1,1-DEE for 15 min. Subsequently, the cells were incubated with 10 μM DCFDA for 15 min. ROS levels were analyzed using a laser scanning confocal microscope (Carl Zeiss, Jena, Germany) with excitation at 488 nm and emission at 515 nm. Additionally, the cells were analyzed by flow cytometry using a BD FACSCalibur system (BD Biosciences, Franklin Lakes, NJ, USA), and data were processed with FlowJo™ software version 10.10 (BD Biosciences, San Jose, CA, USA).

### 2.6. Animal Care and Drug Administration

NSG male mice (4 weeks old) were obtained from the National Immunotherapy Innovation Center (Chonnam National University, Hwasun-gun, Republic of Korea) and housed at the Laboratory Animal Research Center of Chonnam National University. The mice were maintained under a 16 h light/8 h dark cycle at 23 °C with 60% humidity and provided ad libitum access to food and water. Five animals were housed per cage, and their weights were recorded three times a week. All experimental procedures involving live animals complied with the institutional guidelines of Chonnam National University. For tumor induction, SH-SY5Y cells (2 × 10^6^ cells in 100 µL of PBS) were subcutaneously injected into the right flank of each mouse. The mice were divided into the control and treatment groups with five animals in each group. Before tumor induction, the treatment group received 3 doses of 1,1-DEE (110 mg/kg) administered intraperitoneally on alternating days. From day 1 to day 105, the treatment group continued receiving 1,1-DEE injections every other day, whereas the control group received PBS injections. Body weight and tumor volume were assessed throughout the study period, while survival rates were monitored over time.

### 2.7. cBioPortal Analysis

#### 2.7.1. Data Source

Publicly available neuroblastoma transcriptomic data were obtained from cBioPortal (TARGET 2018 dataset: https://www.cbioportal.org accessed on 28 December 2025). Only samples with complete mRNA expression profiles were included. Data were downloaded in TXT format and imported into R (v4.3).

#### 2.7.2. Gene Panel

A curated gene panel was assembled to capture AMPK signaling, inflammation, myogenesis, metabolism, and cachexia biology. AMPK-related genes included PRKAA/PRKAB/PRKAG subunits and STK11. Cachexia-associated genes included MYOD1, MYOG, MSTN, STAT3, IL6, IL1B, TNFSF12, PPARGC1A, SIRT1, MTOR, AKT1, PAX7, CDKN1A, GDF15, BAX, and others previously implicated in muscle wasting and metabolic stress.

#### 2.7.3. Heatmap and Correlation Analysis

Pairwise gene–gene associations were computed using Spearman correlation. Hierarchical clustering was performed using Euclidean distance and Ward’s linkage. Publication-ready heatmaps with dynamic scaling and AMPK vs. cachexia color annotations were generated in R using pheatmap.

### 2.8. Statistical Analysis

All data are presented as mean ± SD, each representing three independent experiments where applicable. Statistical analyses were performed in GraphPad Prism 8.0 and R. Group comparisons were evaluated using one-way ANOVA with Tukey’s post hoc test; when only two groups were compared, an unpaired two-tailed Student’s t-test was applied. Significance thresholds were defined as: *p* < 0.05 (#, *), *p* < 0.01 (##, **), *p* < 0.001 (###, ***), and *p* < 0.0001 (####, ****). Correlation analyses were FDR-adjusted (Benjamini–Hochberg); FDR-adjusted *p* < 0.05 was considered significant.

## 3. Results

**Figure 1:**
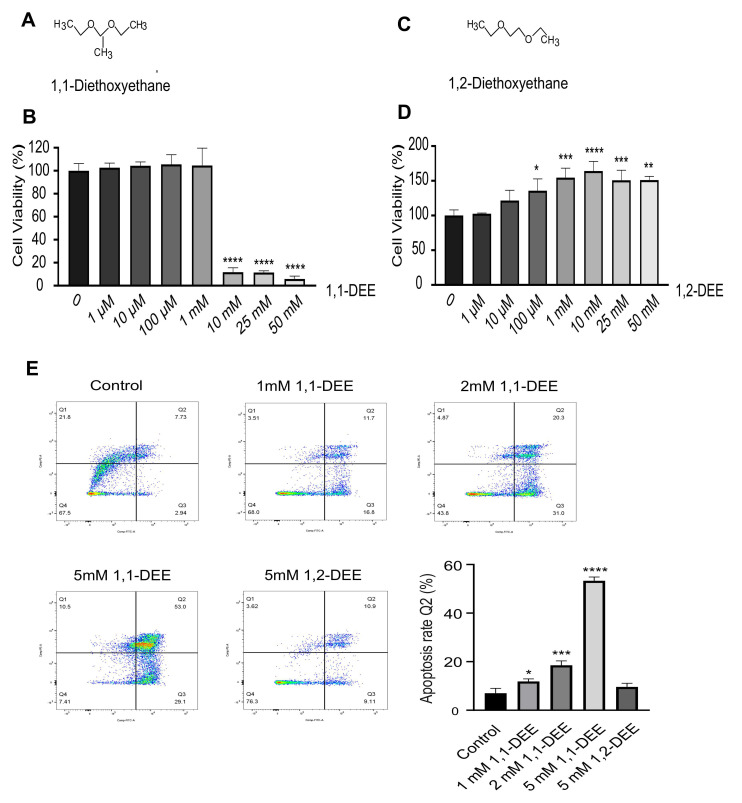
In vitro effect of 1,1-DEE and Apoptosis on SH-SY5Y Cells. (**A**,**B**) Cell viability was assessed by MTT assay. Cells were treated with various concentrations of 1,1-DEE for 24 h. (**C**,**D**) Isomer (1,2-DEE)-treated cells were similarly analyzed for viability. Following treatment, cells were incubated with 10 μL of MTT solution for 2 h. Each experiment was conducted independently in triplicate. (**E**) Apoptosis in SHSY-5Y cells was analyzed by flow cytometry following 24 h of treatment with varying concentrations of 1,1-DEE. The results show a significant dose-dependent increase in the number of apoptotic cells compared to that in the control group and isomer. Data are presented as the mean ± SEM. * *p* < 0.05; ** *p* < 0.01; *** *p* < 0.001; **** *p* < 0.0001 vs. control.

**Cytotoxicity effects 1,1-DEE and its isomer 1,2-DEE in SH-SY5Y cells with induction of Apoptosis:** 1,1-DEE (Figure 1A) inhibited SH-SY5Y cell growth and viability in a dose-dependent manner, as shown in (Figure 1B). At lower concentrations (1 µM to 1 mM), no significant reduction in cell viability was observed compared to that of the control group. However, at higher concentrations (10 mM and 25 mM), 1,1-DEE significantly reduced cell viability respectively. The IC50 value of 1,1-DEE for SH-SY5Y cells at 24 h was 8.63 mM. In contrast, its isomer (1,2-DEE) had no cytotoxic effect on cell viability at various tested concentrations (Figure 1C,D). Based on these findings, a concentration of 5 mM 1,1-DEE was selected for further analysis. We investigated whether 1,1-DEE (1–5 mM) and 1,2-DEE (5 mM)-containing culture affected the proliferation of SH-SY5Y cells. Inverted light microscopy revealed dose- and time-dependent morphological changes and decreased viability following 1,1-DEE treatment; however, no changes were observed when the cells were treated with the isomer (5 mM), as shown in Supplementary Figure S1. To determine whether 1,1-DEE induces apoptosis, SH-SY5Y cells were treated with various concentrations for 24 h and stained with Annexin V and PI. 1,1-DEE induced a concentration-dependent increase in apoptotic cells, as evidenced by an increase in Annexin V^+^/PI^+^ populations (Q2) (Figure 1E). The percentages of late apoptotic or necrotic cells were significantly increased in a dose-dependent manner.

**Figure 2:**
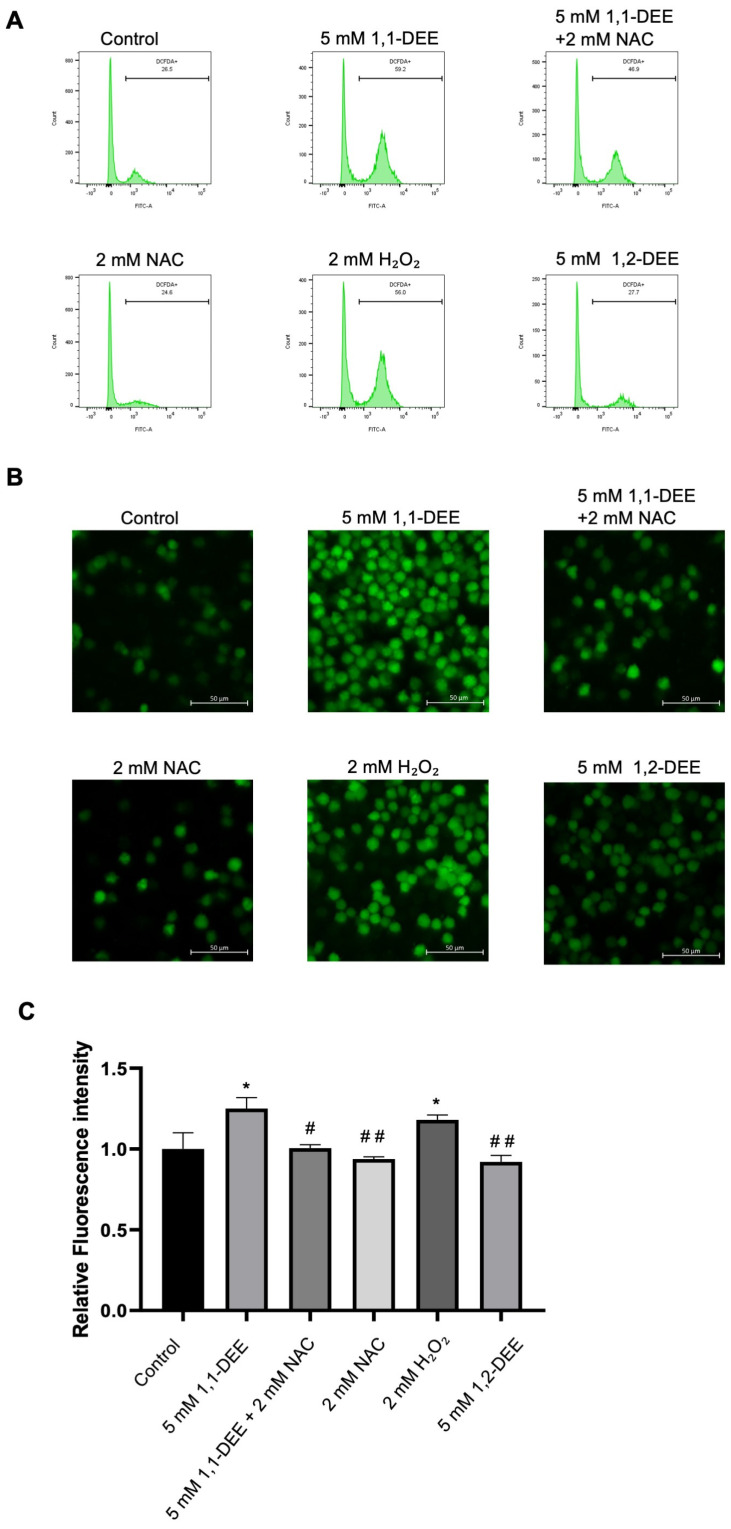
1,1-DEE induced ROS in SH-SY5Y Cells. (**A**) Flow cytometry analysis of ROS production in SH-SY5Y cells was performed after treatment with 1,1-DEE (5 mM) for 15 min and H_2_O_2_ (2 mM) for 30 min, with pretreatment using NAC (2 mM). (**B**) Confocal microscopy (200× magnification) of ROS production and quantitative analysis of ROS levels were performed (**C**). Scale bar: 50 µM. Data are presented as the mean ± SEM. * *p* < 0.05 vs. control. ^#^ *p* < 0.05; ^##^ *p* < 0.01 vs. 1,1-DEE.

**1,1-DEE induced ROS production:** The accumulation of ROS has been implicated in various cellular processes, including proliferation and apoptosis. To assess ROS production, SH-SY5Y cells were treated with 1,1-DEE and stained with the fluorescent probe DCFH-DA. Fluorescence intensity was observed (Supplementary Figure S2), which showed significantly reduced ROS accumulation by 1,1-DEE at 15 min. Treatment with 1,1-DEE (5 mM) markedly induced the accumulation of ROS, which was particularly evident at 15 min, as assessed by both flow cytometry (Figure 2A) and confocal microscopy (Figure 2B) using NAC as ROS scavenger and H_2_O_2_ as positive control. Fluorescence intensity was further observed (Figure 2C) using NAC as a ROS scavenger, H_2_O_2_ and isomer (1,2-DEE) which showed significant ROS regulation. Notably, pretreatment with *N*-acetylcysteine (NAC) for 1 h restored cell viability after 24 h of 1,1-DEE exposure, suggesting that ROS generation contributes to 1,1-DEE cytotoxic effects (Supplementary Figure S3).

**Figure 3:**
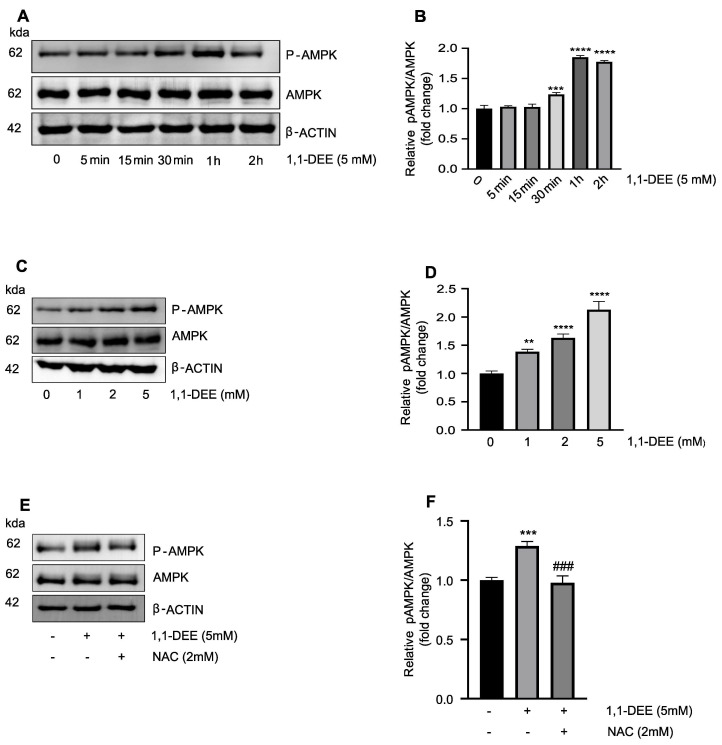
1,1-DEE induced AMPK activation in SH-SY5Y Cells. (**A**,**B**) Western blot analysis of AMPK activation in SHSY-5Y cells was performed. Cells were treated with 1,1-DEE for 5 Mm from 5 min to 2 h, total AMPK and phosphorylated AMPK were measured by Western blotting. (**C**,**D**) Western blot analysis of AMPK activation and phosphorylated AMPK by 1–5 Mm 1,1-DEE for 30 min in SHSY-5Y cells. (**E**,**F**) Cells were pretreated with *N*-acetylcysteine (NAC, 2 mM) for 1 h prior to treatment with 1,1-DEE (5 mM) for 30 min. Data are presented as the mean ± SEM. ** *p* < 0.01; *** *p* < 0.001; **** *p* < 0.0001 vs. control and; ^###^ *p* < 0.001 vs. 1,1-DEE.

**Activation of AMPK by 1,1-DEE:** 1,1-DEE activated AMPK in SH-SY5Y cells in a time and concentration dependent manner, with significant induction observed between 30 min and 2 h (Figure 3A). AMPK activation was also concentration-dependent, with maximal response at different concentrations at 30 min (Figure 3B). Pretreatment with *N*-acetylcysteine (NAC) reduced AMPK phosphorylation following 30 min of exposure to 1,1-DEE, indicating that ROS scavenging attenuates AMPK activity. These results suggest that ROS generation partially contributes to AMPK activation induced by 1,1-DEE.

**Figure 4:**
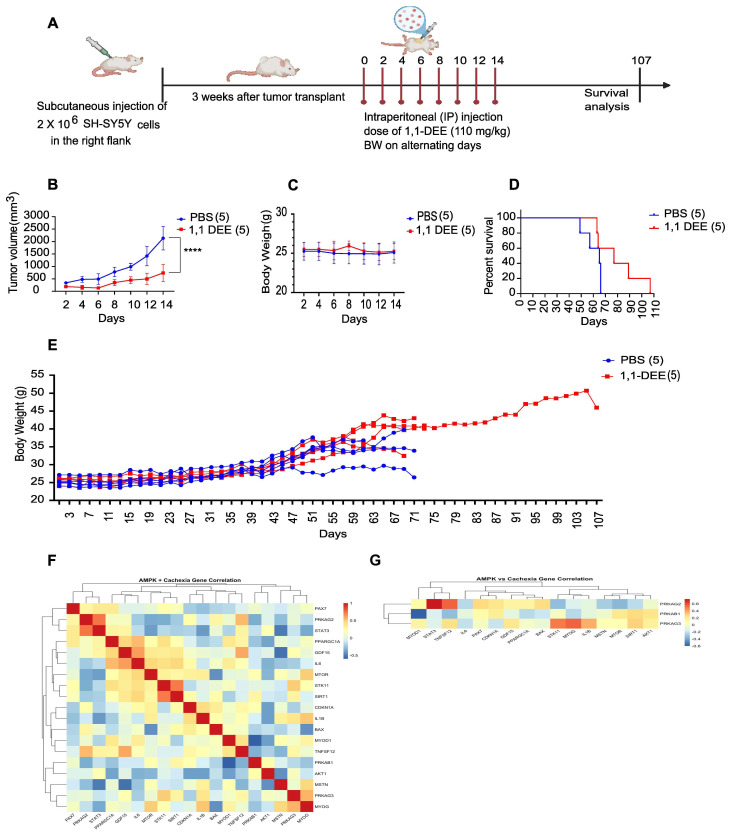
In vivo effect of 1,1-DEE on NSG mice model. (**A**) SH-SY5Y cells (10^7^ cells) were subcutaneously injected into the left thigh of NSG mice, and tumor growth was monitored for 4 weeks. In the treatment group, 1,1-DEE was administered via intraperitoneal injection at a dose of 110 mg/kg on alternate days (days 2, 4, 6, 10, and 12), whereas the control group received PBS. (**B**) Tumor volume was measured over time in the treatment and control groups. (**C**) Body weight during the tumor volume measured. (**D**) Kaplan–Meier survival curves were plotted to compare the survival of mice in the treatment and control groups. (**E**) Body weight measurements were taken at various time points during treatment. Significant differences were observed between the survival of the control and treatment groups. (**F**) Gene Expression Heatmap in TARGET 2018 Cohort Hierarchical clustering of 24 genes involved in AMPK signaling, muscle metabolism, and cachexia across tumor samples. Rows represent genes and columns represent patients. Heatmap color scale indicates z-score normalized expression (blue = low, red = high, white = mean). Dendrograms display co-expression relationships among genes and stratification of patient subgroups. (**G**) AMPK vs. Cachexia Cross-Correlation Heatmap Spearman correlation matrix between AMPK subunits and cachexia-related genes. Red indicates positive correlation, blue negative, and white indicates weak or no correlation. Hierarchical clustering of genes highlights functional modules, and color annotations distinguish AMPK (orange) and cachexia (green) gene categories. **** *p* < 0.0001.

**Suppression of tumor growth in vivo by 1,1-DEE:** To investigate the in vivo antitumor effects of 1,1-DEE, a subcutaneous tumor model was developed using 4-week-old NSG mice. After 3 doses of 1,1-DEE were administered intraperitoneally, SHSY-5Y cells were injected subcutaneously into the mice (Figure 4A). Tumor volumes were measured 3 weeks after injection and every 2 days thereafter. The results showed that 1,1-DEE significantly inhibited tumor growth in vivo. As shown in (Figure 4B), tumor growth curves revealed a significant reduction in tumor volume in the 1,1-DEE-treated group between 21 and 33 days after tumor establishment (*p* < 0.0001). Simultaneously, body weight was monitored (Figure 4C and Supplementary Figure S4), which showed the maintenance of body weight in 1,1-DEE-treated mice. Survival analysis revealed that the 1,1-DEE-treated group exhibited improved survival compared to that of the control group (PBS), as shown in Figure 4D (Kaplan–Meier curve). The median survival for the PBS group was 65 days, whereas the 1,1-DEE-treated group showed a median survival of 77 days, with a hazard ratio of 0.8442 (95% CI: 0.2444 to 2.916). The results suggest that 1,1-DEE may ameliorate cancer cachexia, as indicated by reduced tumor growth and improved survival. (Figure 4E) Body weight of mice was also observed during whole survival study.

**AMPK Activation Defines an Anti-Cachectic Transcriptional State:** Integrated correlation analysis of TARGET-2018 expression profiles revealed that AMPK activation is transcriptionally coupled to an anti-cachectic phenotype (Figure 4F,G). The AMPK catalytic axis, represented by PRKAA2 and its upstream kinase STK11, exhibited strong negative correlations with core cachexia-driving inflammatory mediators, including IL6 (r = −0.84, *p* < 1 × 10^−5^), STAT3 (r = −0.68, *p* = 1.3 × 10^−5^), and GDF15 (r = −0.60, *p* = 1.5 × 10^−3^), indicating suppression of cytokine-driven catabolic signaling (Supplementary Table S1). In parallel, AMPK activation positively aligned with mitochondrial and metabolic preservation programs, as evidenced by significant associations with SIRT1 and PPARGC1A (r = 0.56–0.60, *p* < 0.01; Supplementary Table S1).

In contrast, regulatory AMPK subunits displayed isoform-specific divergence. PRKAB1 showed positive correlations with catabolic and cachexia-associated mediators (TNFSF12, r = 0.52, *p* = 2.8 × 10^−3^) and inverse correlations with myogenic regulators (MYOD1, MYOG), consistent with impaired muscle maintenance (Supplementary Table S1). Collectively, these data establish AMPK activation—particularly via α-subunits—as an intrinsic anti-cachectic transcriptional program, restraining inflammatory signaling while preserving mitochondrial integrity and myogenic potential.

## 4. Discussion

Multidrug resistance remains a central obstacle in the clinical management of Neuroblastoma, particularly in high-risk patients who frequently exhibit poor therapeutic outcomes despite intensive multimodal treatment regimens [18,19,20,21]. Consequently, the identification of novel therapeutic agents capable of overcoming drug resistance and effectively suppressing tumor growth is urgently required. In pursuit of this objective, we investigated a bioactive compound derived from wine for its potential antitumor activity. 1,1-DEE, a volatile compound generated through the chemical interaction of acetaldehyde and ethanol and recognized for its distinctive contribution to wine aroma. Although primarily characterized within the domain of food chemistry, the potential biomedical relevance of this molecule has remained unexplored [22,23]. Here, we present the first mechanistic investigation revealing the anticancer potential of 1,1-DEE.

1,1-DEE elicited a pronounced, dose-dependent reduction in the viability of SH-SY5Y cells (Figure 1B) whereas its structural isomer 1,2-DEE did not elicit a comparable effect This differential response highlights the importance of structural specificity in mediating the cytotoxic activity 1,1-DEE. Consistent with this observation, our previous study delineated the favorable physicochemical profile of 1,1-DEE, characterized by a logP value of 1.15 and a low polar surface area (PSA = 18), parameters indicative of enhanced membrane permeability and efficient intracellular translocation. Notably, in comparison with its isoform 1,2-DEE, Metformin and Resveratrol, 1,1-DEE exhibits more advantageous permeability-associated characteristics, suggesting superior cellular penetration and bioavailability, which may potentiate engagement of cytotoxic signaling cascades culminating in tumor cell death [17,24,25]. Apoptosis induction, as confirmed by 1,1-DEE-FITC/PI staining (Figure 1E), is a key mechanism of action even though tumor cells often upregulate anti-apoptotic factors [26]. The AMPK plays a central role in cellular energy homeostasis and tumor suppression by inhibiting pro-tumorigenic metabolism and inducing cell cycle arrest [27,28]. Our study demonstrated that 1,1-DEE activated AMPK, leading to the inhibition of SH-SY5Y cell growth. The increased phosphorylation of AMPK following 1,1-DEE treatment (Figure 3A,B) correlated with a reduction in cell viability and an induction of apoptosis. Although the role of AMPK is context-dependent, prior studies have demonstrated that its phosphorylation could induce apoptosis in tumor cells while protecting normal cells under energy stress conditions [29]. Moreover, activated AMPK has been found to suppress mTORC1 signaling, promoting apoptosis in SH-SY5Y cells and 6-week-old female BALB/c-nude mice [18]. Our previous work demonstrated that 1,1-DEE activates AMPK in AC16 cardiomyocytes, concomitantly promoting mitochondrial biogenesis through upregulation of PGC-1α and Mitochondrial Transcription Factor A [16]. Although PGC-1α generally maintains mitochondrial homeostasis, accumulating evidence across malignancies—including clear cell renal cell carcinoma [30], musculoskeletal [31], intestinal [32], and epithelial ovarian cancer [33]—indicates that its overexpression can enforce oxidative metabolic reprogramming, intensifying mitochondrial respiration and ROS production. This metabolic shift is consistent with an anti-Warburg effect, characterized by suppression of glycolysis and a reprogramming toward mitochondrial oxidative phosphorylation. The resulting oxidative stress provokes genomic damage and culminates in mitochondria-dependent apoptosis. In this context, 1,1-DEE-mediated activation of AMPK and the consequent mitochondrial remodeling may establish a pro-oxidative metabolic milieu that sensitizes SH-SY5Y cells to apoptotic cell death, thereby providing a compelling mechanistic rationale for its anticancer efficacy.

To further examine the anticancer potential of 1,1-DEE, we evaluated its effects in a NSG Mice model of neuroblastoma. 1,1-DEE treatment significantly reduced tumor growth and volume while improving survival in tumor-bearing mice (Figure 4B–D). Notably, compared to control mice, 1,1-DEE-treated mice maintained a stable body weight (Figure 4E), suggesting the protective effect of 1,1-DEE against cancer cachexia. Cachexia is a multifactorial syndrome characterized by anorexia, weight loss, muscle wasting, and decreased quality of life, which remains under-researched in pediatric oncology [34]. Cachexia not only contributes to treatment-related toxicity but also increases long-term morbidity in children with cancer [35]. A prior study by Carneiro et al. demonstrated that AMPK activation influenced feeding behavior and body weight homeostasis in neuroblastoma N2A cells and Wistar rats [36]. Furthermore, the integrative transcriptomic and functional analyses converge on a coherent model in which AMPK activation orchestrates anti-tumor programs in neuroblastoma through suppression of anabolic signaling, induction of oxidative stress, and engagement of apoptotic and cell-cycle arrest mechanisms. Hierarchical clustering of TARGET 2018 expression profiles revealed coordinated co-expression modules linking pro-inflammatory mediators (IL6, IL1B, STAT3), stress markers (GDF15, CDKN1A, BAX), and metabolic regulators (SIRT1, PPARGC1A), consistent with the well-recognized role of cytokine-driven metabolic dysregulation in tumor biology [37,38] (Figure 4F). (Figure 4G) Cross-correlation mapping further demonstrated that AMPK regulatory subunits (PRKAG2/3) positively associated with inflammatory and stress pathways, whereas subunits such as PRKAB1 exhibit inverse relationships with myogenic regulators (MYOD1, MYOG), highlighting differential engagement of metabolic checkpoints in tumor cells. Collectively, these transcriptional architectures support AMPK as a nodal integrator of nutrient sensing, stress adaptation, and growth restraint [18,39,40].

These findings are concordant with our previous studies demonstrating that compound 1,1-DEE robustly activates AMPK in vivo, conferring systemic metabolic protection. And, in high-fat diet models, 1,1-DEE enhanced mitochondrial ROS production, promoting PTEN oxidation and downstream Akt activation, thereby sensitizing insulin signaling. Consistently, 1,1-DEE potentiated insulin-stimulated Akt activation, reversed palmitate-induced insulin resistance in myoblasts, and improved glucose tolerance and insulin sensitivity in HFD-fed mice [41]. Notably, 1,1-DEE mitigated diet-induced weight gain and hepatic dyslipidemia independently of food intake, supported by transcriptomic reprogramming toward improved metabolic homeostasis. Beyond metabolic disease, 1,1-DEE also conferred protection against ischemic injury in cardiovascular models, underscoring a shared AMPK-centered mechanism of metabolic stabilization across pathological contexts [41]. These observations directly support our current findings that AMPK activation exerts an anti-cachectic function. The ability of compound 1,1-DEE to restore insulin sensitivity, stabilize mitochondrial metabolism, and suppress systemic inflammation in obesity and ischemic models indicates a conserved metabolic-protective program driven by AMPK, which is mechanistically aligned with the suppression of inflammatory–catabolic signaling and preservation of myogenic pathways observed here. Together, these data establish that1,1-DEE activated AMPK which acts as a central metabolic checkpoint that counteracts cachexia-associated energy stress and tissue wasting. Together, these convergent data suggest that controlled AMPK activation redirects energetic stress toward tumor-suppressive outcomes without triggering systemic wasting, supporting a precision metabolic-targeting framework in which AMPK-informed gene signatures may guide patient stratification and therapeutic response. However, further molecular studies are warranted to elucidate this mechanism.

**Figure 5:**
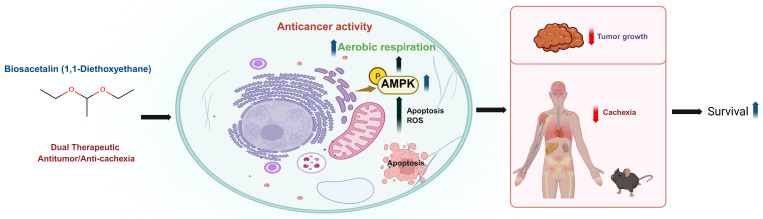
Schematic representation of the proposed mechanism of action of 1,1-DEE. 1,1-DEE activates AMP-activated protein kinase, leading to increased ROS generation, and Apoptosis in SH-SY5Y cells, resulting in tumor growth suppression and prolonged survival in vivo. Concurrently, AMPK activation contributes to attenuation of Cancer Cachexia, supporting systemic metabolic homeostasis.

Collectively, our findings identify 1,1-DEE as a previously unrecognized dual-action therapeutic candidate in which activation of AMP-activated protein kinase functions as a central mechanistic hub (Figure 5), coupling ROS–driven apoptosis in neuroblastoma with attenuation of cancer cachexia, thereby revealing a unified therapeutic paradigm that integrates tumor-selective cytotoxicity with systemic metabolic preservation.

## 5. Conclusions

This study represents the first demonstration that 1,1-DEE robustly induces Apoptosis, concomitantly activates AMPK signaling, and suppresses tumor progression in vivo. Intriguingly, this compound also mitigates systemic metabolic deterioration associated with cancer cachexia and significantly prolongs survival, thereby positioning 1,1-DEE as a promising candidate for the development of mechanism-driven therapeutic interventions against neuroblastoma.

## Data Availability

The original contributions presented in this study are included in the article/Supplementary Material. Further inquiries can be directed to the corresponding author(s).

## Supplementary Materials

The following supporting information can be downloaded at: https://www.mdpi.com/article/10.3390/antiox15040521/s1.
