# Supplementary material for: Biosacetalin (1,1-Diethoxyethane) Prolongs Survival and Alleviates Cachexia in the NSG Mice Bearing Neuroblastoma SH-SY5Y Cells
Source: Antioxidants (Basel). 2026 Apr 21;15(4):521. doi: 10.3390/antiox15040521 (PMC13114220; doi:10.3390/antiox15040521)

Supplementary Figure. S1

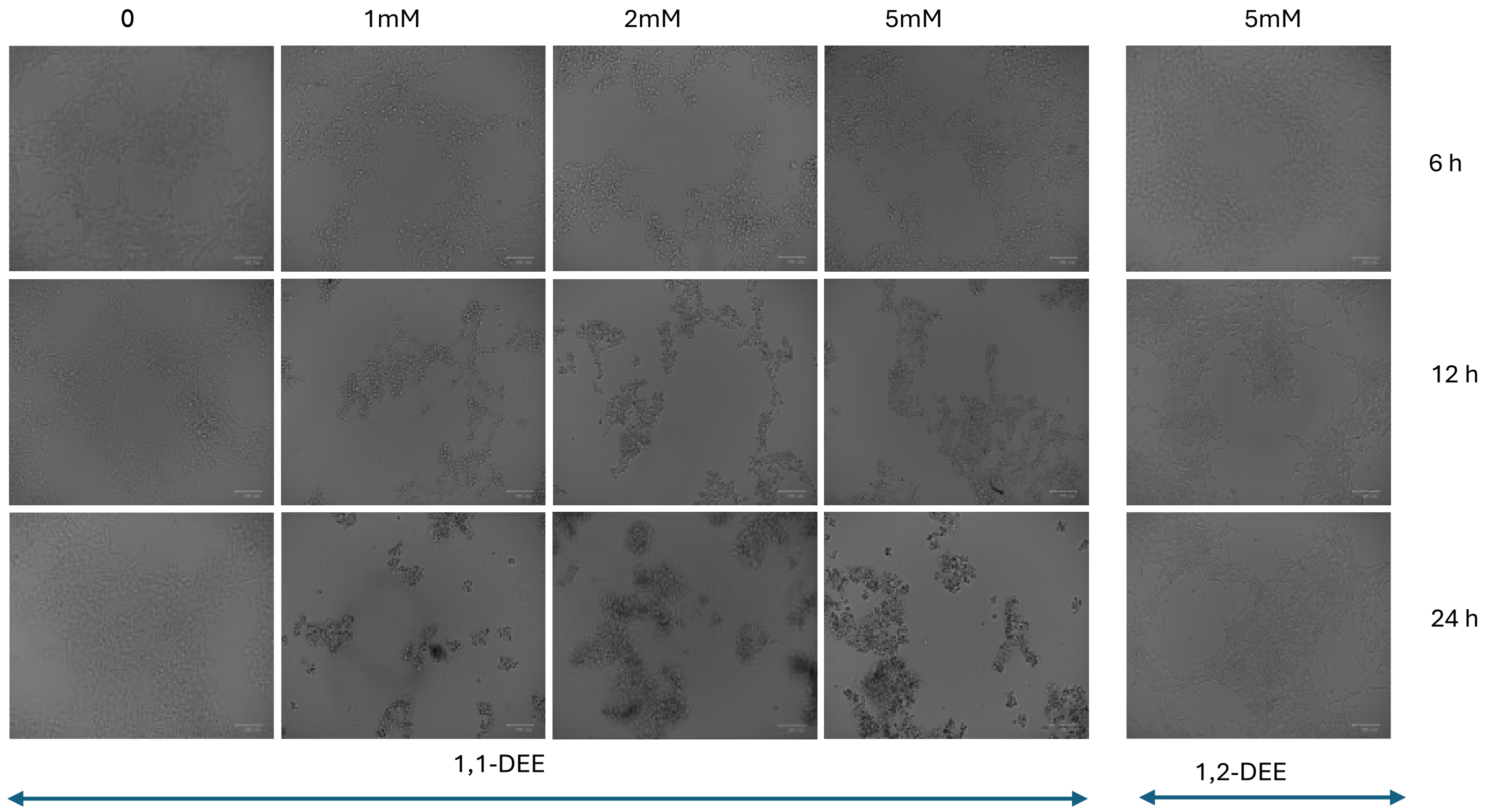

Supplementary Figure. S2

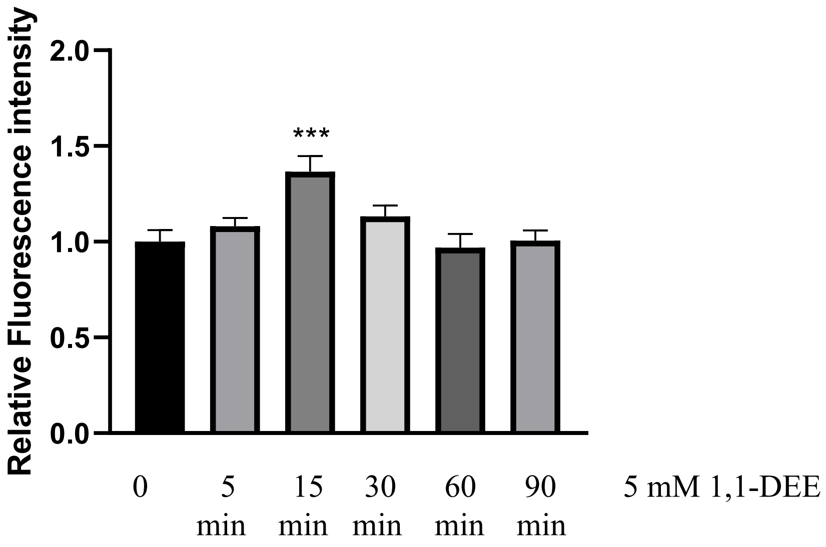

Supplementary Figure. S3

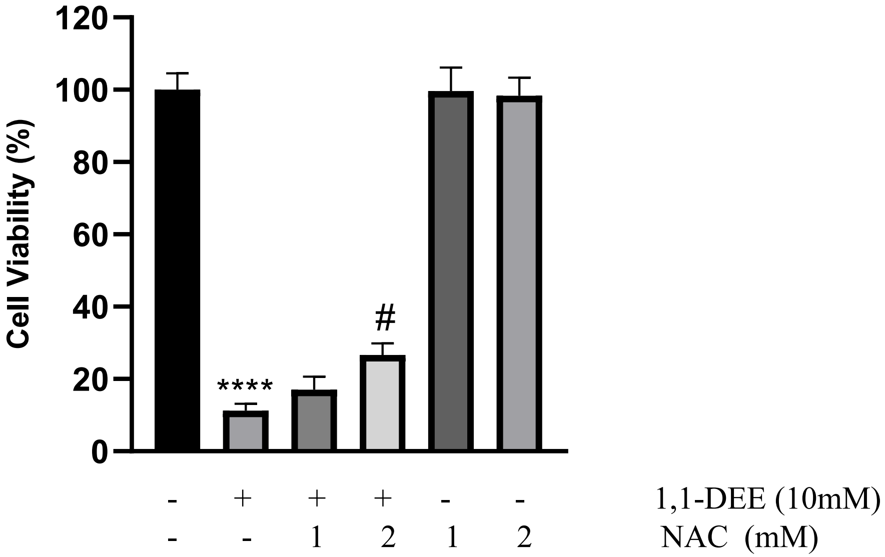

Supplementary Figure. S4

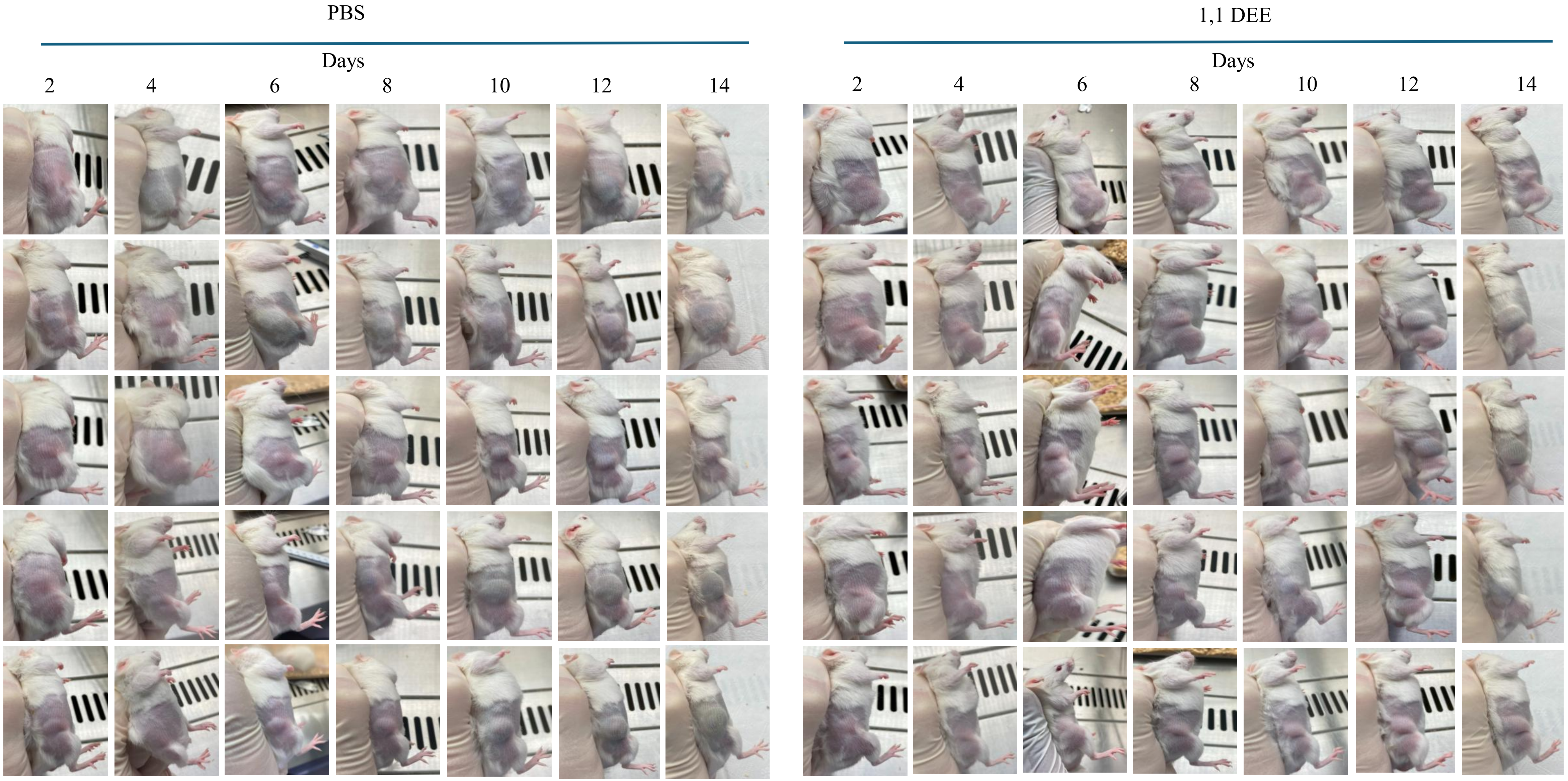

Supplement: Supplementary file 1 [file antioxidants-15-00521-s001.zip › Supplementary file 1.pdf]
